# CKS2 Overexpression Correlates with Prognosis and Immune Cell Infiltration in Lung Adenocarcinoma: A Comprehensive Study based on Bioinformatics and Experiments

**DOI:** 10.7150/jca.63625

**Published:** 2021-10-11

**Authors:** Zhiping Wang, Mengyan Zhang, Yahua Wu, Yilin Yu, Qunhao Zheng, Jiancheng Li

**Affiliations:** Fujian Medical University Cancer Hospital, Fujian Cancer Hospital, Fuzhou, Fujian, China.

**Keywords:** CKS2, lung adenocarcinoma, biomarker, immune infiltration, prognosis, proliferation

## Abstract

**Objective:** Cyclin-dependent kinase regulatory subunit 2 (CKS2) plays a vital role in regulation of the cell cycle and cancer progression. However, the role of CKS2 in lung adenocarcinoma (LUAD) remains unkonwn. Here, we examined the prognostic value and biological functions of CKS2 in LUAD by using omics data of 1,235 LUAD samples from TCGA, GEO, and our own cohort as well as data of *in vitro* experiments.

**Methods:** Kaplan-Meier was conducted to evaluate the prognostic value of CKS2 expression. The association between CKS2 expression level and tumor immune infiltration was explored using the single-sample Gene Set Enrichment Analysis (ssGSEA) and TIMER database. Functional enrichment analyses were performed to annotate the biological functions of CKS2 in LUAD. Furthermore, a series of *in vitro* experiments and immunohistochemistry were performed for validation.

**Results:** CKS2 overexpression was correlated with the advanced stage, TP53 status, PD-L1 expression, and DNA hypomethylation. Moreover, patients with LUAD and high CKS2 expression exhibited poor overall survival. Functional enrichment analysis indicated that CKS2 was involved in cell division, cell cycle, DNA replication. Experiments *in vitro* indicated that CKS2 knockdown decreased the invasion and proliferation of LUAD cells and facilitated their apoptosis. ssGSEA and TIMER analysis revealed a negative correlation between CKS2 expression and the immune cell infiltration**.**

**Conclusions:** In summary, High CKS2 expression was associated with poor prognosis and low levels of infiltrating immune cells in LUAD as well as with malignant phenotypes. Therefore, CKS2 may be a promising prognostic biomarker and therapeutic target in LUAD.

## Introduction

Lung cancer, the most common human malignancy globally, is the leading cause of cancer-related deaths and a huge threat to human health 1. Non-small cell lung cancer (NSCLC) is classified into lung adenocarcinoma (LUAD), lung squamous cell carcinoma, and large cell lung carcinoma according to the histological type. Of these types, LUAD accounts for ~40% of the total number of lung cancer cases annually 2. Because lung cancer initially develops without obvious clinical signs, most patients with NSCLC are not diagnosed until they reach locally advanced or extensive metastatic stages; this complicates treatment, leading to a poor 5-year overall survival (OS) rate of only 18% 3, 4. Despite remarkable advances in the multimodal treatments, including targeted therapies, chemotherapy, as well as radiation therapy, the prognosis of patients with advanced-stage lung cancer remains far from satisfactory. Therefore, identifying effective biomarkers for accurate prediction of the patient's prognosis and/or response to an individualized therapy is important for reducing the mortality rate of patients with lung cancer.

Numerous studies have recently highlighted the importance of the tumor microenvironment in tumor development. In particular, the tumor immune microenvironment (TIM) is considered to be a crucial factor affecting tumor progression and therapeutic response 5. Thus, targeted modulation of TIM parameters may relieve clinical symptoms and improve prognosis of patients with LUAD 6. In addition, recent immunotherapies, such as programmed death-1 receptor (PD-1)/programmed death ligand-1 (PD-L1) pathway blockade, have demonstrated promising effects in various cancers, including NSCLC 7. However, there are few predictive biomarkers to identify patients who can benefit from immunotherapy. Therefore, in the era of precision medicine, it is necessary to elucidate the association between LUAD and the TIM and to identify novel reliable immune-related biomarkers that can predict the prognosis of lung cancer and serve as targets for immunotherapy.

Cyclin-dependent kinase regulatory subunits 1 (CKS1) and 2 (CKS2) belong to a family of highly conserved small (9 KDa) cyclin-dependent kinase (CDK)-binding proteins that play an important role in regulation of the cell cycle 8, 9. Previous studies have shown that CKS2 may play a key role in somatic cell division and early embryonic development 10. Furthermore, multiple lines of evidence suggest that CKS2 is abnormally expressed in several types of tumors, including esophageal cancer, breast cancer, and ovarian cancer, and is involved in tumor progression 11-13. However, the specific biological role and prognostic value of CKS2 in LUAD remain unknown.

In our study, we sought to investigate the relationship between CKS2 expression and the TIM as well as CKS2 function and prognostic value in LUAD. Therefore, we evaluated the prognostic value of CKS2 expression in LUAD using patient data from The Cancer Genome Atlas (TCGA), Gene Expression Omnibus, and our own cohort. To understand the biological functions of CKS2, we performed Gene Ontology (GO) analysis, Kyoto Encyclopedia of Genes and Genomes (KEGG) analysis, and Gene Set Enrichment Analysis (GSEA). Furthermore, *in vitro* functional experiments were conducted to verify the results of the bioinformatic analyses. In addition, the relationship between the methylation status and CKS2 expression was analyzed using bioinformatic tools. Finally, the correlation between CKS2 expression and the level of immune cell infiltration was analyzed using the single-sample Gene Set Enrichment Analysis (ssGSEA) and Tumor Immune Estimation Resource (TIMER) database.

## Materials and methods

### Data sources

RNA-seq fragments per kilobase of transcript per million reads mapped (FPKM) of LUAD samples with clinical data were downloaded from TGCA. RNA-seq samples without clinical data were excluded, and a total of 513 samples were then subjected to further analysis by transforming level 3 HTSeq-FPKM data into transcripts per million (TPM) reads. The GES72094 and GSE31210 databases were downloaded from the National Center for Biotechnology Information (https://www.ncbi.nlm.nih.gov/) for comparison with TCGA dataset. Unavailable or unknown clinical data for individuals were regarded as missing values. The detailed clinical information regarding all samples from TCGA are summarized in **Table 1**. Our set of 98 LUAD specimens and 82 adjacent normal tissues (Tissue chips: HLugA180Su07) was purchased from Outdo Biotech Ltd (Shanghai, China) and used to further verify CKS2 protein expression and its prognostic value in LUAD. The detailed clinical characteristics of our cohort are shown in **Table 3**.

### Analysis of co-expressed and differentially expressed genes

The genes that were co-expressed with CKS2, based on TCGA data, were identified using the R software (v.3.6.2). Spearman correlation was employed to investigate the association between CKS2 and the co-expressed genes. |r| >0.5 and *P* <0.001 represented a significant correlation between CKS2 and the co-expressed genes. Based on the gene expression count of LUAD samples, the patients were divided into high and low expression groups, with the median as the cut-off value. Differentially expressed genes in the HTSeq-TPM dataset were analyzed using the DESeq2 package 14. Log fold change (logFC) >1 and corrected *P* <0.01were set as the threshold of differential genes. The data of the differentially expressed and co-expressed genes were visualized using heat maps and volcano plots.

### GO and KEGG enrichment analyses

Metascape (http://metascape.org), an excellent gene list analysis tool for gene annotation and analysis, can be used to perform GO and KEGG enrichment analysis 15. Genes that were co-expressed with CKS2 (|r| > 0.5, *P* < 0.001) and differentially expressed between the high and low expression groups (adjusted *P* value < 0.01, |logFC| >1) simultaneously were regarded as CKS2 related genes, which were uploaded to the Metascape online tool for GO and KEGG enrichment analyses. *P* <0.01, minimum count >3, and enrichment factor >1.5 were considered significant.

### GSEA

To assess the significance of differences in the signaling pathway components between groups with high and low *CKS2* expression, GSEA of *CKS2* expression data from TCGA was performed using clusterProfiler package (3.8.0) in R software 16. The enrichment was classified as significant if |NES| > 1 and *P* value < 0.05.

### Immune infiltration analysis using ssGSEA and TIMER

ssGSEA was performed using the GSVA package in R to classify marker gene sets in a single sample with physiological and biological function and chromosomal localization and to predict the level of infiltration of immune cells in tumor samples based on the signature genes of 24 types of immunocytes 17. The Spearman correlation between CKS2 expression and 24 types of immune cells was evaluated, and the association between the infiltration of immune cells and *CKS2* expression was studied. Significant results (*P* value < 0.01, |r|> 0.1) were displayed using a lollipop plot. Furthermore, the TIMER online tool (https://cistrome.shinyapps.io/timer/) 18 was applied to validate the correlation between *CKS2* expression and the level of immune cell infiltration.

### Analysis of methylation and copy number variation levels

The methylation and copy number variation (CNV) data for *CKS2* were downloaded from the cBioPortal web platform (https://www.cbioportal.org/) 19. *CKS2* expression in different *CKS2* CNV groups was compared using the Kruskal-Wallis test. The Spearman correlation test was used to explore the association between the *CKS2* methylation level and expression. In addition, the SMART web platform (http://www.bioinfo-zs.com/smartapp/) 20 was used to analyze and compare the methylation level of *CKS2* through pan-cancer analysis of TCGA data.

### Immunohistochemistry

Immunohistochemistry was conducted to determine CKS2 protein expression and its prognostic value. The tissue chips were stained by immunohistochemistry with anti-human CKS2 (Abcam, United Kingdom), followed by HRP secondary antibody and DAB treatment. Then, the samples were observed under a microscope and photographed for further analysis.

The staining results were scored based on the staining intensity and fraction of positive cells (staining score = staining intensity score × fraction of positive cell score). The staining intensity was scored as 0 (negative), 1 (weak), 2 (medium), or 3 (strong). The fraction of positive cells was scored as 0 (≤10%), 1 (11%-25%), 2 (26%-50%), 3 (51%-80%), or 4 (≥81%). The staining score of each sample was evaluated independently by two experienced pathologists.

### Cell lines and transfection

Human lung carcinoma A549 and H1299 cell lines were purchased from the American Type Culture Collection (Manassas, VA, USA). A549 cells were cultured in F12K medium supplemented with 10% fetal bovine serum (FBS), 100 mg/mL streptomycin, and 100 U/mL penicillin at 37 °C in an atmosphere of 95% air and 5% CO_2_. H1299 cells were cultured in RPMI 1640 medium supplemented with 10% FBS, 100 mg/mL streptomycin, and 100 U/mL penicillin at 37 °C in an atmosphere of 95% air and 5% CO_2_. For selective knockdown of *CKS2*, we used three small interfering RNAs (siRNAs si-1, si-2, and si-3) and negative control (NC) siRNA obtained from GenePharma (D010003; Shanghai, China). The siRNA sequences were as follows:

- CKS2 si-1 sense: 5'-CUUGGUGUCCAACAGAGUCUATT-3';
- CKS2 si-1 antisense: 5'-UAGACUCUGUUGGACACCAAGTT-3';
- CKS2 si-2 sense: 5'-GAGUCUAGGCUGGGUUCAUUATT-3';
- CKS2 si-2 antisense: 5'-UAAUGAACCCAGCCUAGACUCTT-3';
- CKS2 si-3 sense: 5'-CCACAUAUUCUUCUCUUUAGATT-3';
- CKS2 si-3 antisense: 5'-UCUAAAGAGAAGAAUAUGUGGTT-3'.

### Quantitative real-time polymerase chain reaction (qRT-PCR)

Total RNA was extracted from transfected cells using a Trizol RNA extraction kit (Huamaike, Fangshan, Beijing, China) based on the manufacturer's instructions. Next, a reverse transcription kit (HengFei Biotech, China) was used to reverse-transcribe the RNA into complementary DNA (cDNA). Subsequently, qRT-PCR was performed to measure the expression of *CKS2* using SYBR® Premix Ex Taq™ II (TaKaRa, Japan). The calculation of relative gene expression levels was performed using the 2^-ΔΔCt^ method. The primers used were as follows:

CKS2-forward: 5'-TTAGTCTCCGGCGAGTTGTT-3', CKS2-reverse: 5'-CACCAAGTCTCCTCCACTCC-3', GAPDH-forward: 5'-GAAGGTGAAGGTCGGAGTC-3', GAPDH-reverse: 5'-GAAGATGGTGATGGGATTTC-3'.

### Western blotting

Total protein was extracted from A549 or H1299 cells using the RIPA extraction reagent (Beyotime, Shanghai, China). To determine protein concentration, the bicinchoninic acid assay (Thermo Fisher Scientific, Waltham, MA, USA) was used. Cell lysate aliquots containing 20 mg of total protein from each experimental condition were resolved by sodium dodecyl sulfate‐polyacrylamide gel electrophoresis in 10% polyacrylamide gels and transferred onto polyvinylidene difluoride membranes (Millipore, Bedford, MA, USA). After blocking in 5% bovine serum albumin (Sigma, USA), the membranes were incubated with an anti-CKS2 antibody (1:500, Abcam, United Kingdom) overnight at 4 °C, washed with Tris-buffered saline + Tween-20, and incubated with a horseradish peroxidase-coupled secondary antibody for 1 hour at room temperature (25 °C). Protein bands were visualized using chemiluminescence.

### Cell counting kit-8 (CCK-8) assay

The A549 and H1299 lung carcinoma cells transfected with NC or siRNA against CKS2 were seeded into a 96-well plate at a density of 5,000 cells/well. Cell viability was measured at 0, 12, 24, 36, 48, 72 and 96 hours using the CCK-8 assay kit (MCE, Shanghai, China). Briefly, 10 μL of CCK-8 reagent (MCE, Shanghai, China) was added daily to each well in the 96-well plates and incubated at 37 °C for 1.5 hours. The optical density value was measured at 450 nm using a microplate reader (Infinite® M1000 PRO, TECAN, Switzerland).

### Cell apoptosis

To measure the extent of apoptosis, transfected A549 and H1299 cells were seeded in 6-well plates at 3 × 10^5^ cells/well and cultured for 48 hours. The cells were washed with cold phosphate-buffered saline and stained using an Annexin V-FITC apoptosis detection kit according to the manufacturer's instructions (US Everbright R Inc, Suzhou, China). A FACSCalibur flow cytometer (BD Biosciences, Mountain View, CA, USA) was used to assess the effects of CKS2 silencing on LUAD cell apoptosis. All experiments were carried out three times.

### Cell invasion assay

Invasion assays were conducted using the 24-well transwell chambers with 8‐μm pores (Costar, Corning, New York, NY, USA). The inserted top side of the chambers was coated with 100 μL of diluted (1:5) Matrigel film (BD Biosciences, Bedford, MA, USA) in a 24-well plate at 37 °C for 4 hours. The process chamber containing 200 μL of the serum-free medium was inoculated with 5×10^4^ cells, whereas 600 μL of the medium with 10% FBS (AusGENEX, Australia) was added to the lower chamber. Following incubation at 37 °C for 48 hours, a cotton swab was used to wipe off the uninvaded cells in the upper chamber, and the cells that invaded to the bottom of the membrane were fixed in 4% paraformaldehyde for 20 minutes and stained with 0.4% crystal violet solution for 15 minutes. Subsequently, the stained cells were studied and photographed under a microscope (Leica, USA) using a 100× magnification. All experiments were repeated three times.

### Statistical analysis

All statistical analyses were conducted using R software (v3.6.2) and Prism 8.0 (GraphPad Software Inc., San Diego, CA, USA). The Wilcoxon signed-rank and Wilcoxon rank-sum tests were used to analyze *CKS2* expression in paired and non-paired samples, respectively. The correlation between clinical pathological features and *CKS2* expression was analyzed using Kruskal-Wallis test, Wilcoxon rank-sum test and Wilcoxon signed-rank test. The survival analysis was performed using the Kaplan-Meier method, and the differences between groups were assessed using the log-rank test. Univariate and multivariate analyses using Cox proportional hazard modeling were performed to estimate the risk of death. Potential confounders included sex, age, clinical stage, and chemotherapy cycles. Student's *t*-test was carried out for comparisons between each group in experiments *in vitro*. Effects were considered significant if *P* < 0.05.

## Results

### *CKS2* expression is upregulated in LUAD based on TCGA database

**Figure 1:**
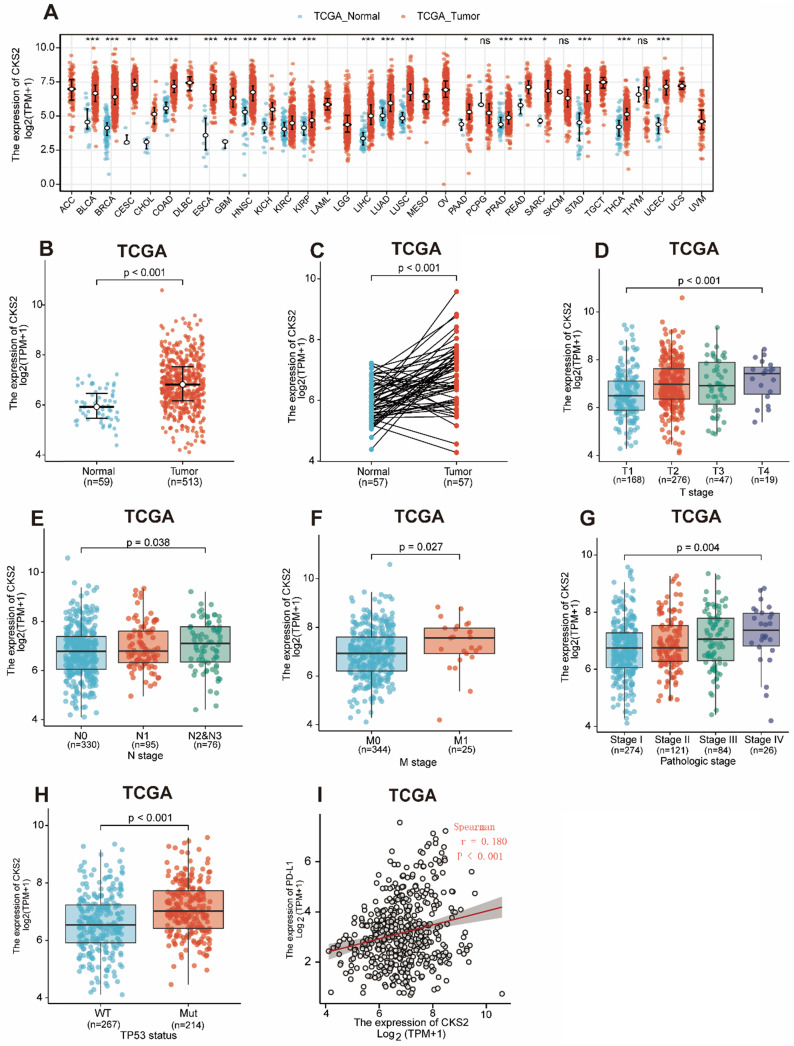
***CKS2* expression levels in LUAD and other types of human cancers from TCGA data. (A)** *CKS2* expression levels in different tumor types from TCGA database. **(B)** *CKS2* expression levels in LUAD and normal tissue. **(C)** The expression of *CKS2* in LUAD and its paired adjacent tissues. **(D)** Association of *CKS2* expression with the T stage in LUAD. **(E)** Association of CKS2 expression with the N stage in LUAD. **(F)** Association of *CKS2* expression with the M stage in LUAD. **(G)** Association of *CKS2* expression with the pathological stage in LUAD. **(H)** Association of *CKS2* expression with the *TP53* status in LUAD. **(I)** Correlation between PD-L1 and *CKS2* expression levels in LUAD.

Statistical analysis of paired tumor and normal adjacent samples as well as of non-paired samples using the Wilcoxon rank-sum and Wilcoxon singed-rank tests, respectively, showed that *CKS2* expression was significantly higher in cancer tissues than in normal tissues (**Figure 1A, C**; *P* < 0.001). To further evaluate *CKS2* expression in human cancers, we examined pan-cancer RNA-seq data from TCGA database. The differential expression of *CKS2* between the tumor and adjacent normal tissues is shown in **Figure 1B**.

Notably, increased expression of *CKS2* was significantly correlated with topographical distribution (**Figure 1D**, *P* < 0.001), lymph node metastasis (**Figure 1E**, *P* = 0.038), distant metastasis (**Figure 1F**, *P* = 0.027), pathological stage (**Figure 1G**, *P* = 0.004), *TP53* status **(Figure 1H**, *P* < 0.001) and PD-L1 expression (**Figure 6I**,* P* < 0.001, r = -0.180).

### High *CKS2* expression is associated with adverse outcomes in LUAD

A total of 513 eligible samples were obtained from TCGA. The patient characteristics associated with these samples, including TNM stage, pathological stage, gender, race, smoking history, *TP53* status and age are listed in** Table 1**.

**Figure 2:**
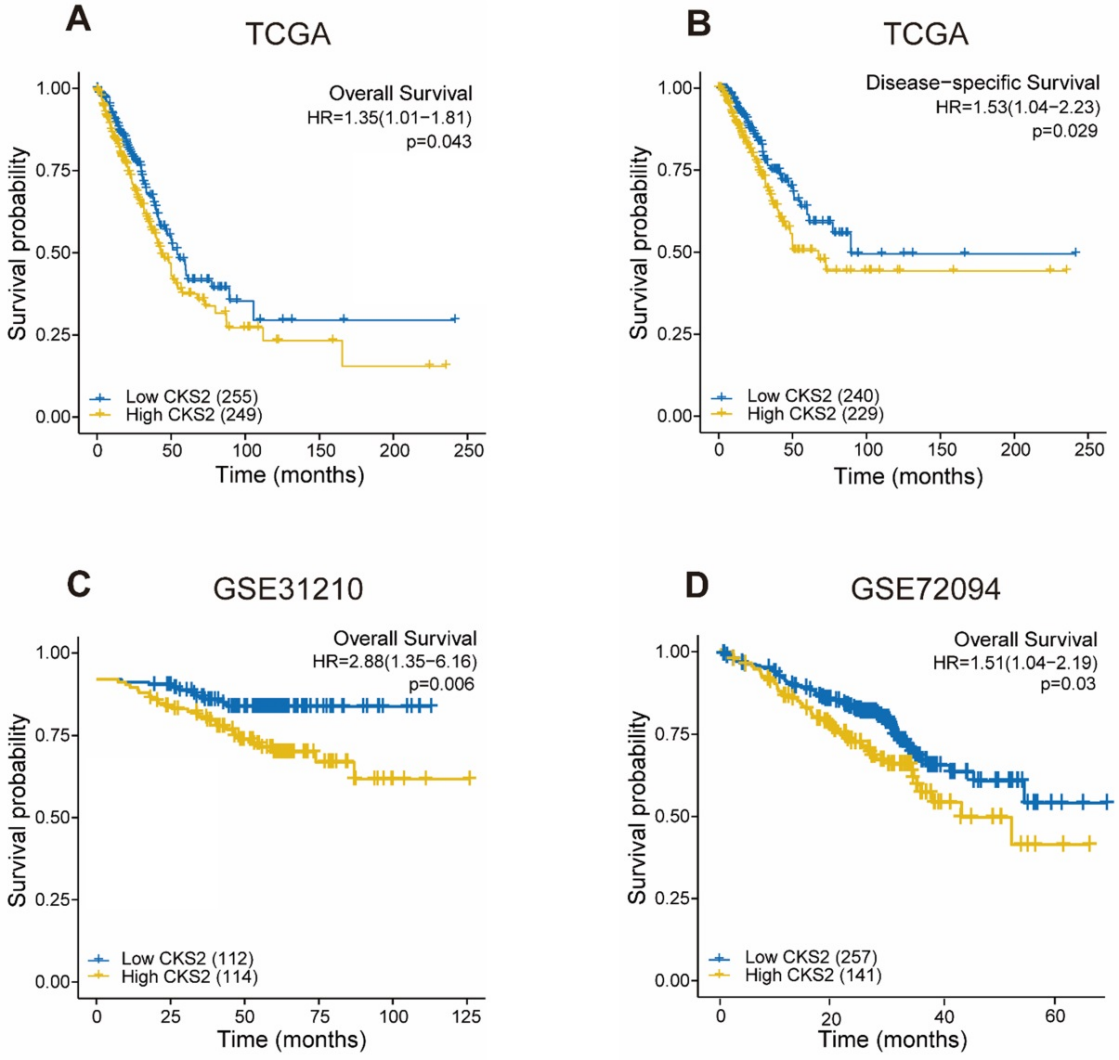
** Prognostic value of *CKS2* expression in LUAD. (A)** Kaplan-Meier curves of OS of patients with high or low *CKS2* expression in TCGA cohort (n = 504). **(B)** Kaplan-Meier curves of DSS of patients with high or low *CKS2* expression in TCGA cohort (n = 469). **(C)** Kaplan-Meier curves of OS of patients with high or low *CKS2* expression in the GSE31210 cohort (n=226). **(D)** Kaplan-Meier curves of OS of patients with high or low *CKS2* expression in the GSE72094 cohort (n =398).

As shown in Figure 2, disease-free survival was significantly poorer in patients with high *CKS2* expression than in those with low *CKS2* expression (**Figure 2B**, *P* = 0.029). A similar result was observed in OS analysis (**Figure 2A**, *P* = 0.046). To verify the relationship between *CKS2* expression and OS, the GSE72094 and GSE31210 datasets were analyzed. In the GSE72094 dataset, patients with high *CKS2* expression had worse prognoses than patients with low *CKS2* expression (**Figure 2D**, *P* = 0.029). Similar results were observed in the GSE31210 dataset (**Figure 2C**, *P* < 0.001).

Univariate analysis revealed that high *CKS2* expression was associated with poor OS (hazard ratio [HR] = 1.353; 95% confidence interval [CI]: 1.009-1.815, *P* = 0.043). Other clinicopathological variables that were correlated with poor OS included T stage (*P* = 0.003), N stage (*P* < 0.001), and pathological stage (*P* = 0.002) (**Table 2**). Upon further multivariate analysis, *CKS2* expression remained independently correlated with OS (HR = 1.403; 95% CI: 1.035-1.902, *P =* 0.029) and pathological stage.

### Functional analysis of *CKS2*-related genes

**Figure 3:**
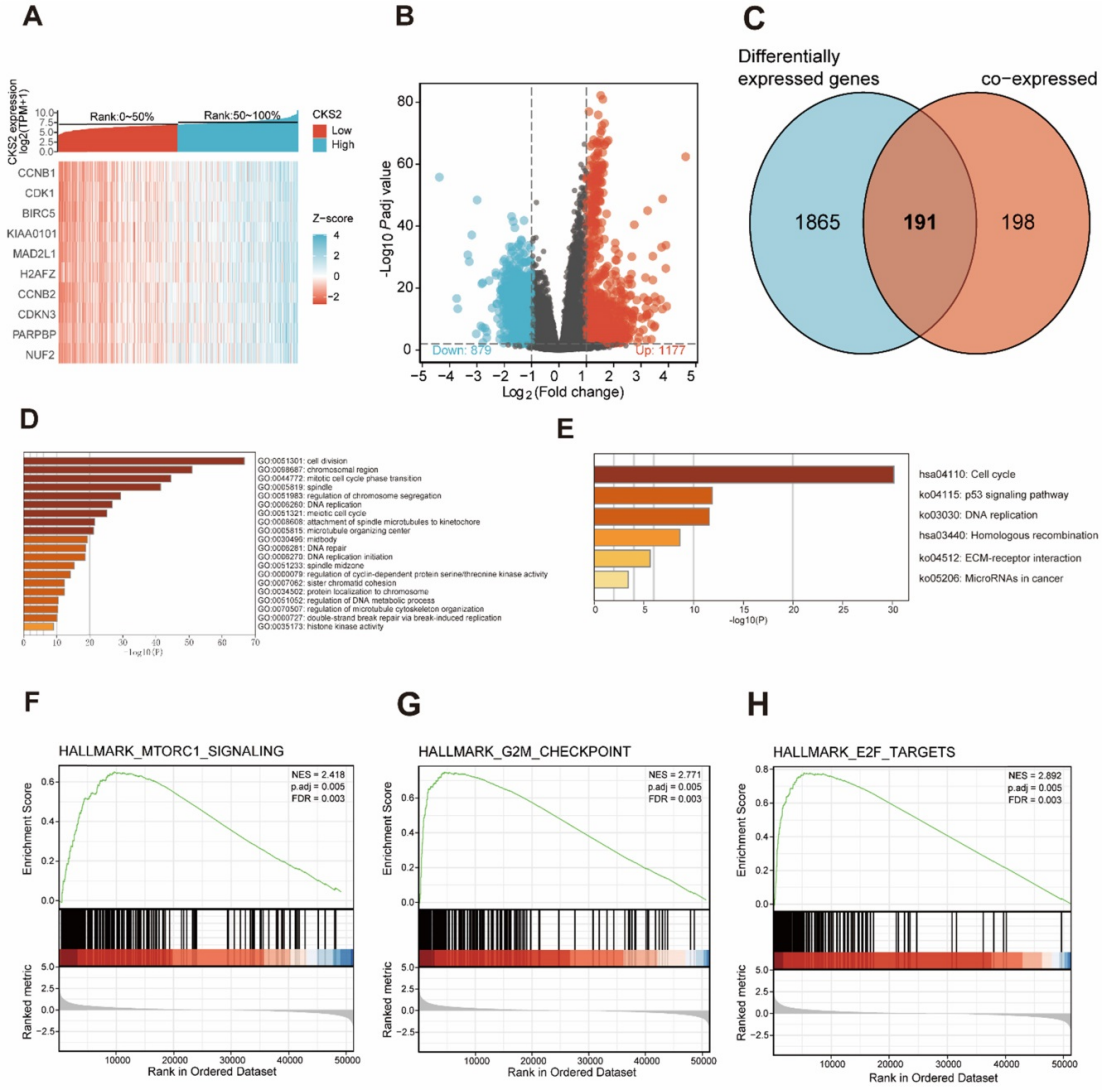
** Functional enrichment of *CKS2* in LUAD. (A)** Heatmap of top 10 genes co-expressed with *CKS2*. **(B)** Volcano plot of differentially expressed genes. **(C)** Venn diagram of 191 CKS2 related genes. **(D, E)** GO and KEGG enrichment analyses of *CKS2* related genes. **(F)** Enrichment of genes involved in the mTORC1 signaling pathway, as revealed by GSEA. **(G)** Enrichment of genes involved in the G2M checkpoint, as revealed by GSEA. **(H)** Enrichment of genes in the E2F targets pathway, as revealed by GSEA.

A total of 389 genes were found to be co-expressed with *CKS2* (|r| > 0.5, *P* < 0.001). Among these, 329 genes were positively correlated with *CKS2*, while 60 genes were negatively with correlated *CKS2* expression. The top 10 co-expressed genes were positively correlated with *CKS2* (**Figure 3A**). Besides, a total of 2056 differentially expressed genes meeting the cut-off criteria (adjusted *P* value < 0.01, |logFC| > 1), including 1177 upregulated genes and 879 downregulated genes, were identified using DESeq2 package in the R software. The differentially expressed genes are illustrated by volcano plot in **Figure 3B.**

Analysis of co-expressed and differentially expressed genes identified 191 *CKS2*-related genes (**Figure 3C**), which were selected for simultaneous GO and KEGG enrichment analyses using the Metascape online tool. The top 20 GO enrichment terms, including three functional groups: biological process group (14 GO terms), molecular function group (5 GO terms), and cellular component (1 GO term), are shown in **Figure 3D**. We found that the *CKS2*-related genes were mainly involved in cell division, regulation of cell cycle, DNA replication, and chromosome. KEGG pathway analysis revealed that *CKS2*-related genes were enriched in the cell cycle, p53 signaling pathway, DNA replication and homologous recombination (**Figure 3E**). Together, these results suggest that the upregulation of *CKS2* potentially regulates LUAD progression via cell division, cell cycle, DNA replication, p53 signaling pathway, and homologous recombination.

### Potential CKS2-related multiple pathways identified through GSEA

GSEA of low and high *CKS2* expression datasets revealed significant differences (|NES| > 1.5, adjusted *P* < 0.01, false discovery rate < 0.25) in enrichment in the MSigDB Collection (h.all.v7.0.symbols.gmt). Additionally, GSEA results showed that the G2M checkpoint, MTORC1 signaling, and E2F targets were activated in patients with high *CKS2* expression (**Figure 3F-H**).

### Correlation between CKS2 expression and immune cell infiltration

**Figure 4:**
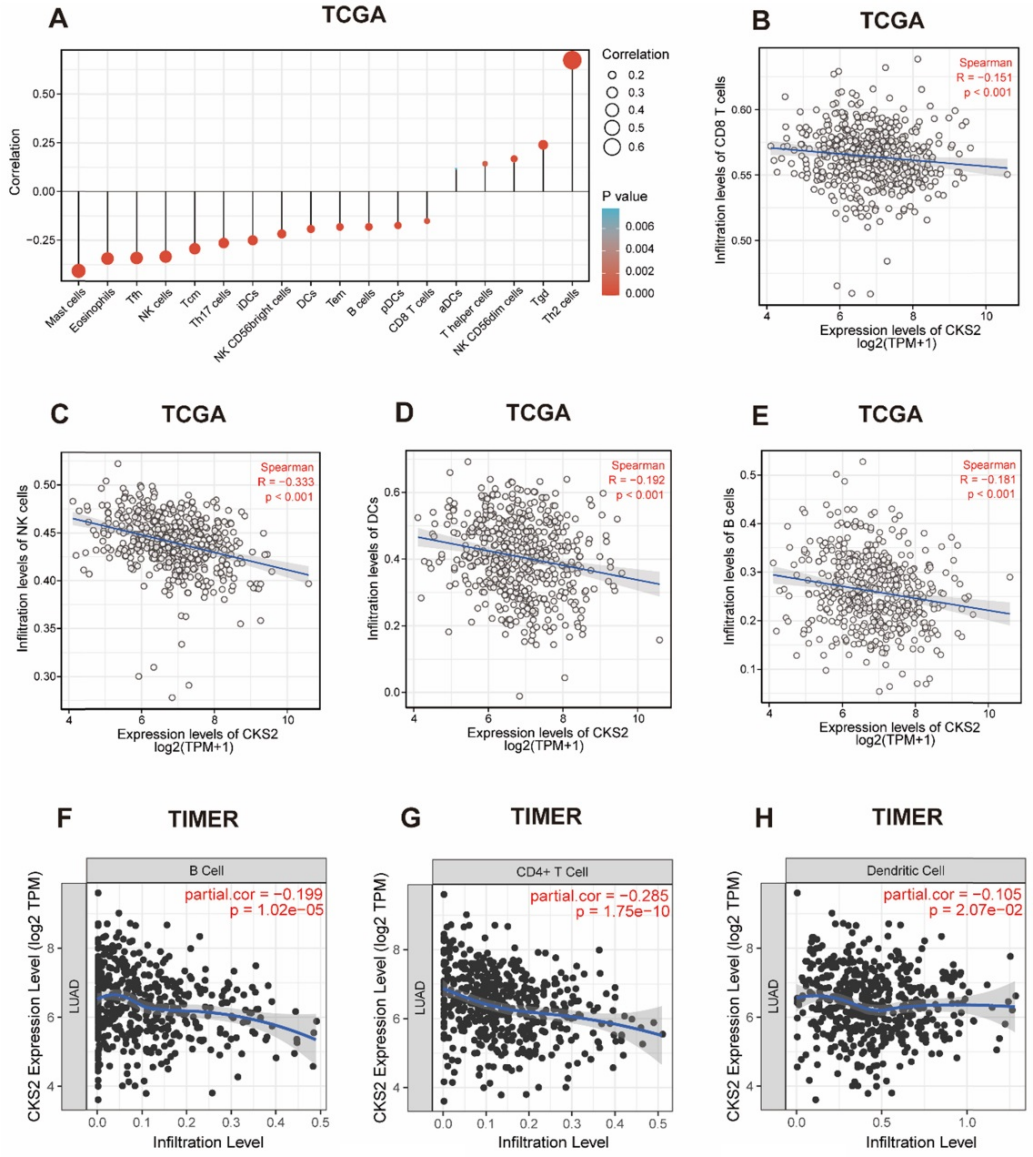
** The correlation of CKS2 expression with the level of immune cell infiltration in LUAD. (A)** Correlation between the level of immune cell infiltration and CKS2 expression by ssGSEA. **(B-E)** CKS2 expression shows a significant negative correlation with infiltrating levels of CD8+ T cells (B), NK cells (C), activated DCs (D), and B cells (E) based on ssGSEA. **(F-H)** CKS2 expression negatively correlates with infiltrating levels of B cells (F), CD4+ T cells (G) and Dendritic cells (J) via TIMER database.

We further applied ssGSEA to analyze the relationship between *CKS2* expression and immune cell infiltration levels. As shown in **Figure 4A**, the numbers of activated dendritic cells (DCs), T helper (Th) cells, natural killer (NK) CD56dim cells, T gamma delta cells, and T helper 2 (Th2) cells were positively correlated with *CKS2* expression. The strongest positive correlation was observed between the number of Th2 cells and CKS2 expression (*P* < 0.001, r = 0.674). In contrast, the numbers of CD8^+^ T cells (**Figure 4B**;* P* < 0.001, r = -0.15), NK cells (**Figure 4C**;* P* < 0.001, r = -0.33), DCs (**Figure 4D**;* P* < 0.001, r = -0.19), and B cells (**Figure 4E**;* P* < 0.001, r = -0.18) were negatively correlated with *CKS2* expression. Similar negative correlations between CKS2 expression and cell numbers were noted for other immune cell subsets, including mast cells, eosinophils, T follicular helper cells, T central memory cells, T effector memory cells, plasmacytoid DCs, and T helper 17 cells (all *P* < 0.05). Furthermore, analyses using the TIMER database revealed that the *CKS2* expression level was negatively correlated with the infiltration of B cells (**Figure 4F**;* P* < 0.001, r = -0.199), CD4^+^ T cells (**Figure 4G**;* P* < 0.001, r = -0.285), and DCs (**Figure 4H**; *P* = 0.02, r = -0.105).

### Hypomethylation is correlated with the overexpression of *CKS2*

**Figure 5:**
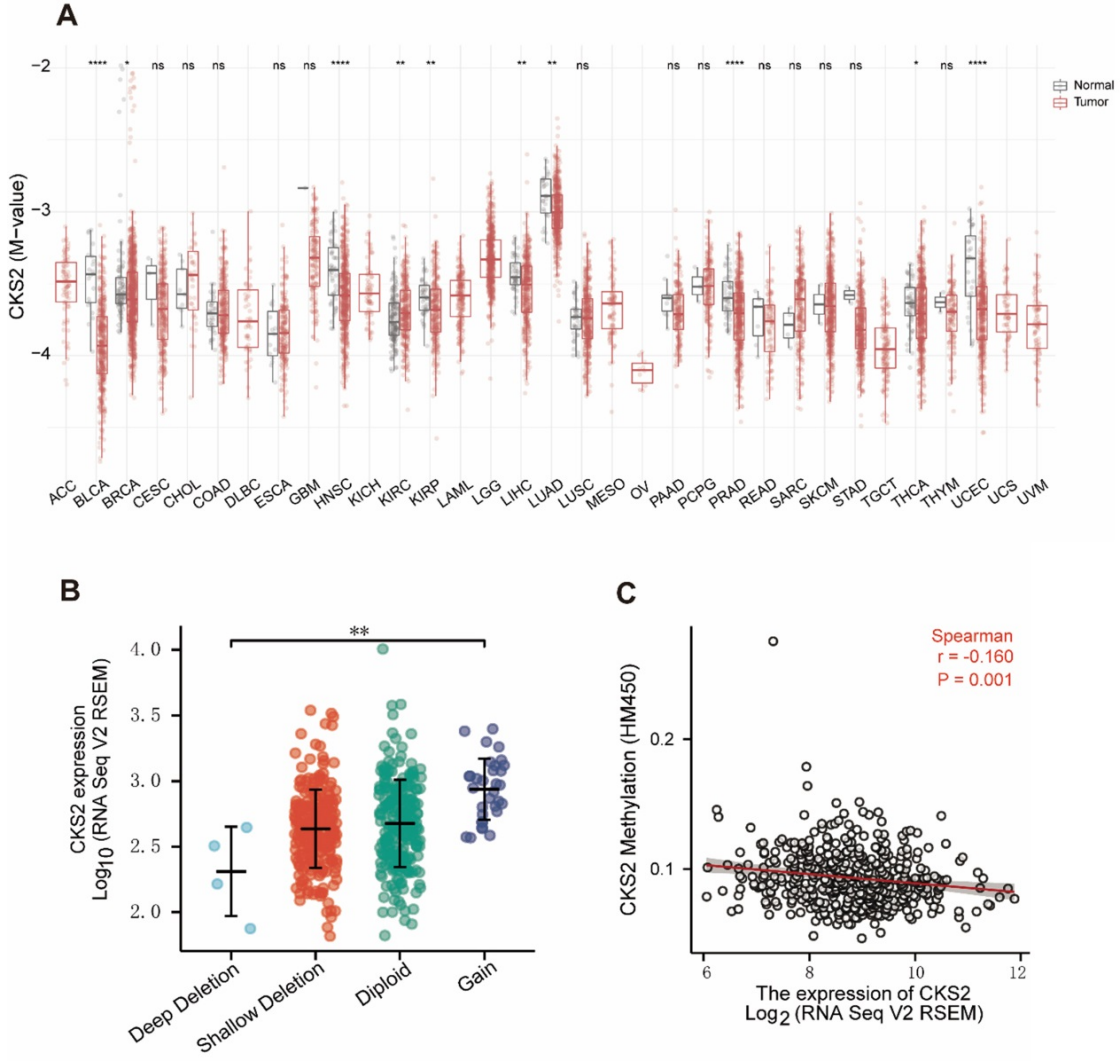
** Copy number variation (CNV) and methylation of *CKS2* in LUAD. (A)** Methylation levels of *CKS2* in pan-cancer and normal tissues according to TCGA data. **(B)** The expression level in different CNVs of LUAD. **(C)** Correlation between *CKS2* methylation and its expression level.

Analysis of the relationship between *CKS2* expression and CNV and methylation levels showed that patients who had a high number of *CKS2* copies exhibited high *CKS2* expression. However, only 6% of the patients exhibited copy number amplification, indicating that CNVs may not be the main driver of high *CKS2* expression (**Figure 5B**). In contrast, *CKS2* expression was negatively correlated with *CKS2* methylation levels (**Figure 5C**). Moreover, *CKS2* methylation levels in the tumor tissues in LUAD, head and neck squamous cell carcinoma, bladder urothelial carcinoma, kidney renal clear cell carcinoma, kidney renal papillary cell carcinoma, liver hepatocellular carcinoma, prostate adenocarcinoma, and uterine corpus endometrial carcinoma were significantly lower than those in the adjacent normal tissues (**Figure 5A**).

### Immunohistochemical validation of the prognostic value of CKS2

**Figure 6:**
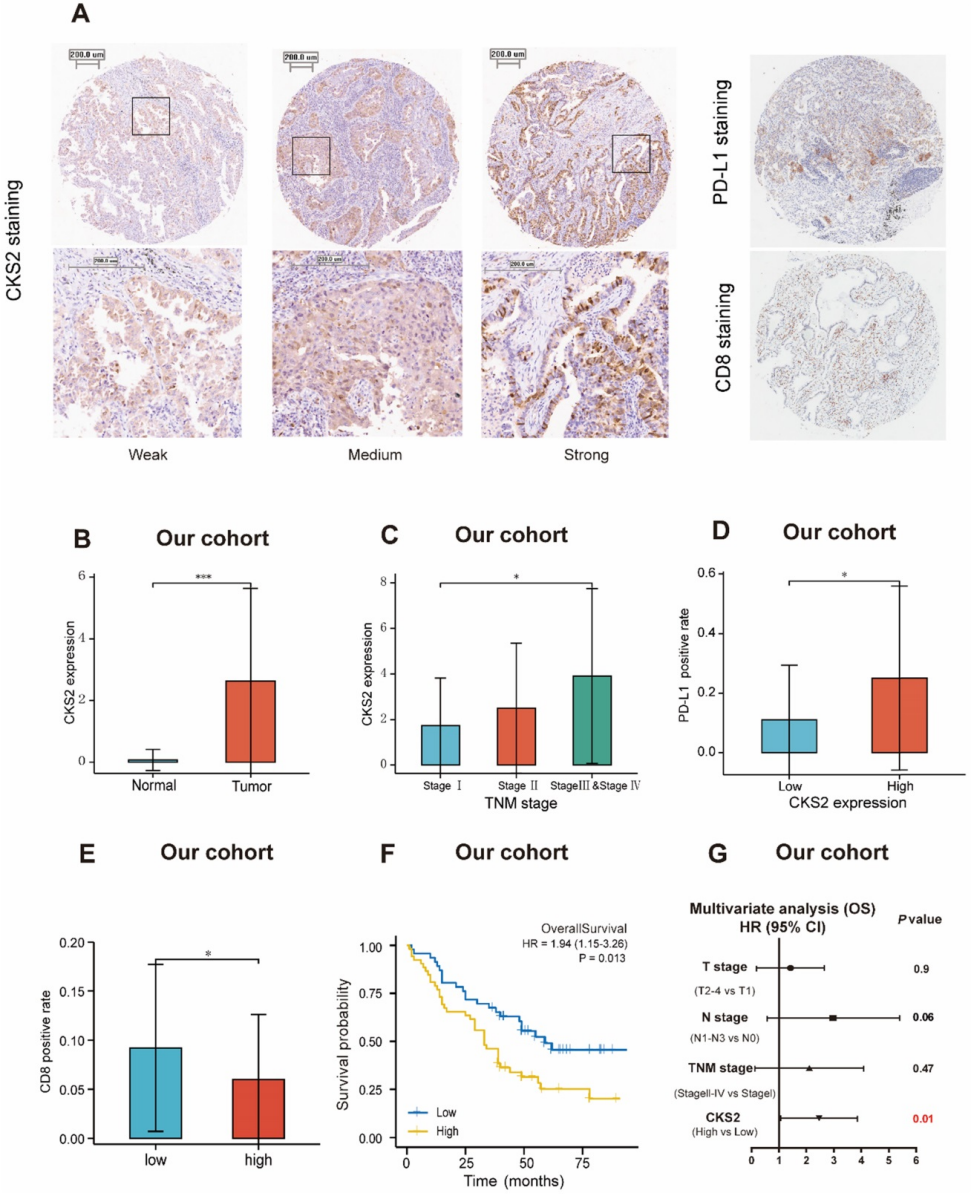
** Immunohistochemical validation of *CKS2* protein expression. (A)** Different intensities of immunohistochemical *CKS2* staining, PD-L1 staining and CD8 staining in samples from patients with LUAD. **(B)** *CKS2* expression in LUAD and normal tissues. **(C)** Association of *CKS2* expression with the TNM stage in LUAD. **(D)** Association of *CKS2* expression with the PD-L1 positivity rate in LUAD. **(E)** Association of *CKS2* expression with the CD8 positivity rate in LUAD. **(F)** Kaplan-Meier curves of OS of patients with high or low *CKS2* expression (n = 98). **(G)** Multivariate Cox regression analysis indicating that high expression of *CKS2* is an independent risk factor for OS in LUAD. (*P < 0.05, **P < 0.01, ***P < 0.001).

The prognostic value of CKS2 protein expression was verified by immunohistochemistry. Images of different immunohistochemical staining intensities of CKS2 (weak, medium, and strong), PD-L1 staining and CD8 staining are shown in **Figure 6A**. Compared with that in adjacent normal tissues, the CKS2 staining score in LUAD tissues was significantly higher (**Figure 6B**, *P* < 0.001). Furthermore, high CKS2 expression was significantly correlated with the TNM stage (**Figure 6C**, *P* < 0.05), high PD-L1 expression (**Figure 6D**, *P* < 0.05) and low CD8 positive rate (**Figure 6E**, *P* < 0.05), consistent with the result from TCGA data. The Kaplan-Meier analysis revealed that the OS was shorter in LUAD patients with high CKS2 expression than in patients with low CKS2 expression (**Figure 6F**, *P* = 0.016). Furthermore, multivariate Cox regression analysis revealed that high CKS2 expression was an independent risk factor of LUAD (**Figure 6G**,* P* = 0.01). This finding was similar to the results of TCGA data analysis.

### Knockdown of *CKS2* in LUAD cell lines

**Figure 7:**
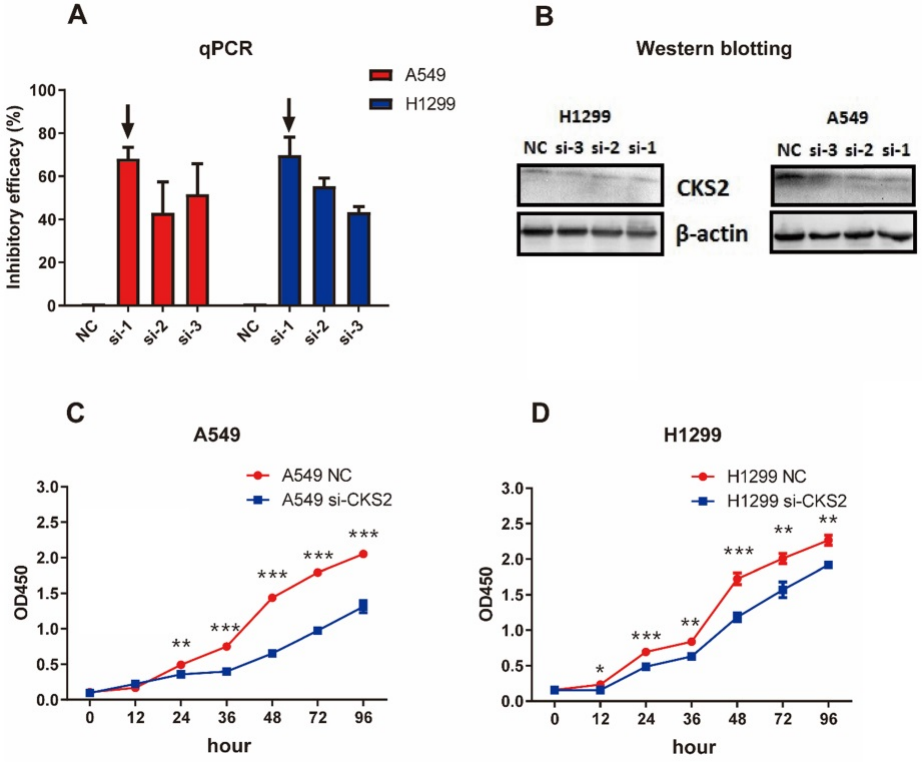
** Silencing *CKS2* expression by treatment with an anti-*CKS2* siRNA attenuates LUAD cell proliferation. (A)** Relative *CKS2* mRNA expression levels in A549 and H1299 LUAD cells transfected with si-1, si-2, si-3 siRNAs against *CKS2* and NC siRNA, as determined by qRT-PCR. **(B)** Relative CKS2 protein expression levels in A549 and H1299 cells transfected with si-1, si-2, si-3 siRNAs against *CKS2* and NC siRNA, as revealed by western blotting. **(C, D)** Decreased survival of A549 and H1299 cells transfected with si-1 siRNA against *CKS2,* as shown by the CCK-8 assay (*P < 0.05, **P < 0.01, ***P < 0.001 vs. cells treated by NC siRNA).

Three siRNAs (si-1, si-2, and si-3) were used to silence *CKS2* in pilot experiments; qRT-PCR results revealed that si-1 had the best inhibitory efficacy among the three siRNAs in both A549 and H1299 cell lines (**Figure 7A**). Furthermore, compared to si-2 and si-3, si-1 induced a stronger decrease in CKS2 protein expression in A549 and H1299 cell lines, according to western blotting results (**Figure 7B**). These data demonstrated that si-1 was effective in inhibiting CKS2 expression in LUAD cell lines; therefore, si-1 was selected for subsequent experiments.

### Knockdown of *CKS2* inhibits the malignant phenotype of LUAD cells

**Figure 8:**
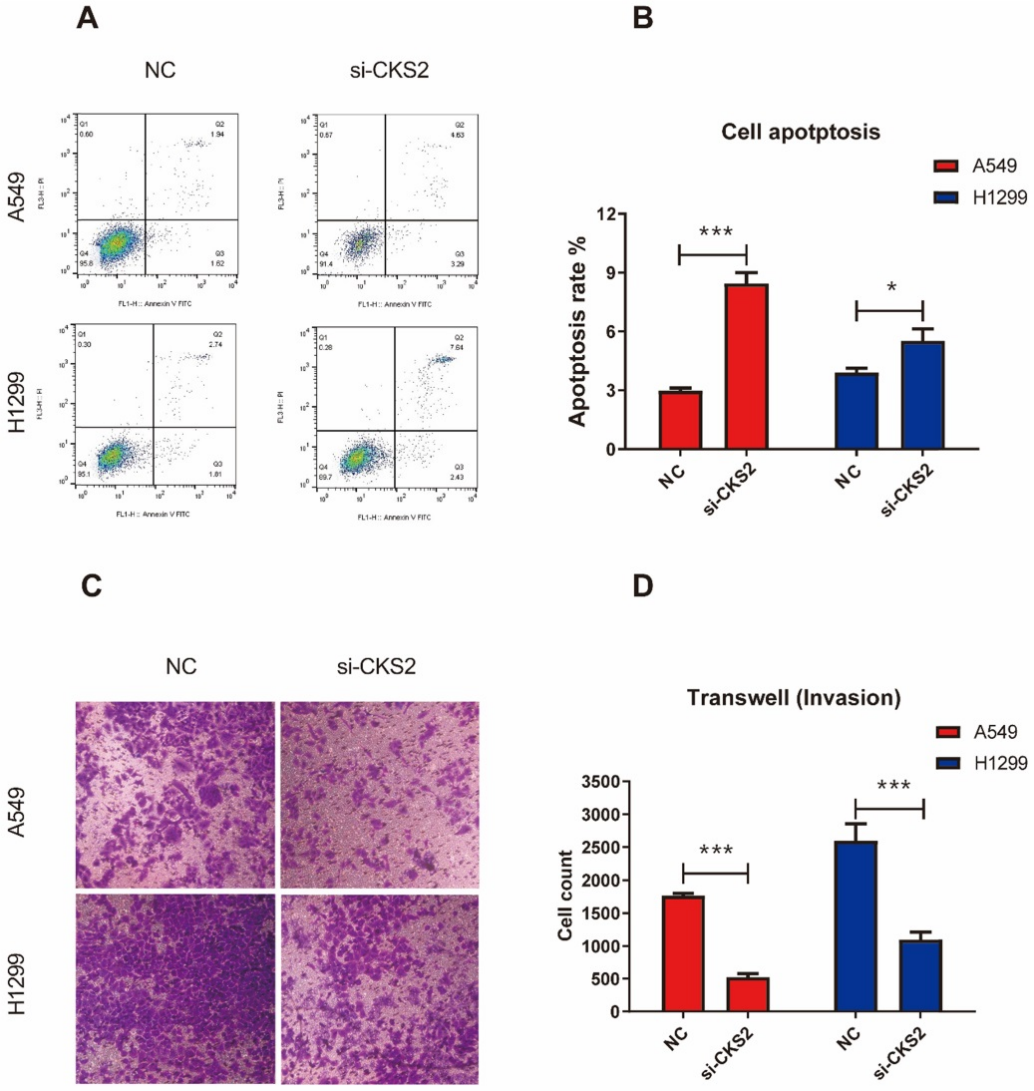
***CKS2* knockdown enhances cell apoptosis and suppressed cell invasion in LUAD cells. (A, B)** *CKS2* knockdown promoted apoptosis of A549 and H1299 LUAD cells according to flow cytometry analysis. **(C, D)** Invasion potential of A549 and H1299 cells transfected with si-1 siRNAs against *CKS2* and NC siRNAs, as determined by the transwell invasion assay (*P < 0.05, **P < 0.01, ***P < 0.001).

To investigate how the inhibition of *CKS2* expression would affect LUAD cells, several *in vitro* experiments were performed. The CCK-8 assay revealed that repressing *CKS2* expression notably decreased the survival of A549 and H1299 cells compared to that in the respective NC groups (**Figure 7B, C**). Apoptosis measurements using flow cytometry demonstrated that compared to the control, a 48-hour exposure to *CKS2* siRNA significantly promoted apoptosis of A549 and H1299 cells (**Figure 8A, B**; A549: *P* < 0.001; H1299:* P* < 0.05). Additionally, the transwell assay revealed that silencing of *CKS2* expression significantly decreased the invasion abilities of A549 and H1299 cells (**Figure 8C, D**; A549: *P* < 0.001; H1299:* P* < 0.001). Taken together, these results indicate that *CKS2* knockdown remarkably suppressed the malignant phenotypes of LUAD cells.

## Discussion

Given the poor OS rate of LUAD 21, it is important to accurately determine its prognosis. In this study, we have demonstrated that *CKS2* tends to be expressed at a higher level in LUAD tissues than in adjacent normal tissues and that *CKS2* expression is negatively correlated with *CKS2* methylation levels. Furthermore, we found that high *CKS2* expression is correlated with various clinicopathological variables and poor OS in LUAD. In addition, functional enrichment analysis indicated that *CKS2* expression is involved in the cell cycle, DNA replication, p53 signaling pathway, MTORC1 signaling pathway, E2F targets, and homologous recombination. Furthermore, high *CKS2* expression is correlated with a low infiltration level of CD8^+^ T cells, CD4+ T cells, NK cells, B cells, and DCs. Overall, our study explored the potential role of CKS2 in tumor pathogenesis and demonstrated its value as a potential LUAD biomarker.

Previous studies have shown that CKS2 is overexpressed in multiple tumors, such as esophageal cancer, breast cancer, and ovarian cancer 11-13. Consistent with these previous reports, we found that in TCGA data, *CKS2* expression levels are higher in LUAD and other tumors than in normal tissues. Moreover, we showed that high *CKS2* expression is significantly correlated with *CKS2* hypomethylation. DNA methylation is an epigenetic mechanism that plays an important role in regulating gene expression by inhibiting transcription factors that bind to DNA or by recruiting proteins related to gene repression 22. A large number of evidences revealed that DNA methylation acts to suppress transcription, and it is reasonable to propose that DNA hypomethylation contributes to tumorigenesis through the activation of oncogenes^23^. This suggests that CKS2 hypomethylation may be the potential cause behind the high expression of CKS2.

*CKS2* is highly expressed in LUAD and is associated with poor prognosis. We found that in TCGA and Gene Expression Omnibus, patients with high *CKS2* expression had worse OS than patients with low *CKS2* expression. In fact, *CKS2* expression was an independent prognostic factor of OS. This result was also verified by multivariate analysis of the LUAD data in our cohort, which indicated that *CKS2* expression is an independent prognostic factor of LUAD. In addition, we found that *CKS2* expression was correlated with clinicopathologic variables, including T stage, N stage, M stage, and pathological stage.

CKS2, a member of the CKS protein family, binds to CDK and plays an important role in regulation of the cell cycle 8. Previous studies have demonstrated that CKS2 binds to the cyclin B1-CDK1 protein kinase, which is essential for mitosis, and that promoting the G2 cell cycle transition induces cells to progress past the G2 phase of the cell cycle 24, 25. Besides, there is sufficient evidence that high CKS2 expression promotes tumor proliferation and invasion in multiple tumors, including hepatocellular carcinoma and colon cancer 26, 27. However, the biological functions of CKS2 in LUAD remained unknown. In this study, our functional enrichment analyses revealed that CKS2 was closely involved in proliferation-associated biological processes, including cell cycle, cell division, and DNA replication. To further verify the bioinformatic analysis results, a series of *in vitro* experiments were conducted in A549 and H1299 LUAD cells. Our experiments indicated that silencing *CKS2* expression remarkably suppressed the proliferation and invasion of LUAD cells as well as promoted apoptosis, which is consistent with our bioinformatic prediction. Notably, in TCGA data, *CKS2* was highly expressed in patients with *TP53* mutations. Furthermore, KEGG analysis suggested that CKS2 was significantly enriched in the p53 signaling pathway. Previous studies have shown that *CKS2* transcription is downregulated by p53 28. Thus, *TP53* mutations may be potential drivers of the high *CKS2* expression in LUAD. Given its role in LUAD progression, CKS2 could be a potential target of antitumor therapy against LUAD.

Cancer progression is affected not only by mutations but also by immune cells in the tumor microenvironment 29-31. Some studies have shown that the expression of tumor genes could impact the type and proportion of immune cells in the tumor microenvironment 32-34. More and more studies have confirmed the important role of mTOR in the regulation of innate and acquired immunity 35. Given that the alteration of mTORC1 signaling pathways was identified by GSEA analysis, we further performed ssGSEA to explore the association between CKS2 expression and immune cell infiltration in LUAD. Interestingly, patients with high *CKS2* expression displayed low infiltration levels of B cells, CD8^+^ T cells, CD4+ T cells, NK cells, and DCs, indicating that high *CKS2* expression may suppress innate immunity and adaptive immunity in the tumor microenvironment, based on the results from TCGA, TIMER and our cohort. Some reports have suggested that low infiltration levels of B cells, CD8^+^ T cells, CD4+ T cells, NK cells, and DCs are related to the poor prognosis of LUAD 36-38. Therefore, we postulate that immunosuppression related to high *CKS2* expression is an important determinant of poor prognosis in LUAD. Recently, Immunotherapies of advanced NSCLC with antibodies against PD-1 or PD-L1 has shown significant clinical efficacy 39, but there are few predictive biomarkers to identify patients who can benefit from immune checkpoint inhibitors (ICIs) treatment. For unselected patients, the response rate of immunotherapy ranges from 14% to 20% 40. More and more evidence showed that patients with high infiltration level of immune cells, in particular CD8+ T cell, indicated better response of ICIs treatment, compared with tumors without lymphocytes infiltration 41. Given that the negative correlation with low level of immune cells infiltration, *CKS2* can serve as a biomarker for providing a reference for the application of immunotherapy in LUAD. Taken together, these results suggest that *CKS2* is a potential biomarker for evaluation of the immune microenvironment.

Although this study improves our understanding of the role of CKS2 in LUAD, it has some limitations. While bioinformatic analyses revealed that *CKS2* expression is negatively correlated with immune cell infiltration, further studies are needed to investigate the specific role of CKS2 in the tumor microenvironment. In addition, more *in vivo* and *in vitro* experiments will be necessary to investigate the specific mechanisms and pathways of action of CKS2 in LUAD.

## Conclusion

In conclusion, our study illustrated that high *CKS2* expression was related to poor prognosis. Moreover, *CKS2* overexpression was correlated with a low level of immune cell infiltration in LUAD. Additionally, *in vitro* experiments verified the biological roles of CKS2 in LUAD and established that CKS2 likely plays an essential role in the malignant phenotype and may be a promising prognostic biomarker for LUAD.

## Figures and Tables

**Table 1:** Demographic and clinical characteristics of LUAD patients in TCGA

Characteristics	level	Overall
n		513
T stage (%)	T1	168 (32.9%)
	T2	276 (54.1%)
	T3	47 (9.2%)
	T4	19 (3.7%)
N stage (%)	N0	330 (65.9%)
	N1	95 (19.0%)
	N2	74 (14.8%)
	N3	2 (0.4%)
M stage (%)	M0	344 (93.2%)
	M1	25 (6.8%)
Pathological stage (%)	Stage I	274 (54.3%)
	Stage II	121 (24.0%)
	Stage III	84 (16.6%)
	Stage IV	26 (5.1%)
Gender (%)	Female	276 (53.8%)
	Male	237 (46.2%)
Race (%)	Asian	7 (1.6%)
	Black or African American	52 (11.7%)
	White	387 (86.8%)
Smoker (%)	No	74 (14.8%)
	Yes	425 (85.2%)
*TP53* status (%)	Mut	241 (47.4%)
	WT	267 (52.6%)
Age (%)	≤65	238 (48.2%)
	>65	256 (51.8%)
CKS2 expression	Low	257 (50.1%)
	High	256 (49.9%)

**Table 2:** Univariate and multivariate analyses of overall survival according to CKS2 expression, after adjusting for other potential predictors in TCGA

Characteristics	Total (N)	HR (95%CI) Univariate analysis	P value Univariate analysis	HR (95%CI) Multivariate analysis	P value Multivariate analysis
T stage (T2-T4 vs. T1)	501	1.668 (1.184-2.349)	0.003	1.161 (0.808-1.670)	0.419
N stage (N1-3 vs. N0)	492	2.606 (1.939-3.503)	<0.001	1.328 (0.823-2.143)	0.245
Pathological stage (Stage II-IV vs. Stage I)	496	2.975 (2.188-4.045)	<0.001	2.257 (1.350-3.773)	0.002
Gender (Male vs. Female)	504	1.060 (0.792-1.418)	0.694		
Age (>65 vs. ≤65)	494	1.228 (0.915-1.649)	0.171		
Smoker (Yes vs. No)	490	0.887 (0.587-1.339)	0.568		
*TP53* status (Mut vs. WT)	499	1.254 (0.936-1.680)	0.130		
*CKS2* (High vs. Low)	504	1.353 (1.009-1.815)	0.043	1.403 (1.035-1.902)	0.029

**Table 3:** Demographic and clinical characteristics of LUAD patients in our cohort

Characteristics	Level	Overall
n		98
T stage (%)	T1	20 (20.4%)
	T2	50 (51.0%)
	T3	21 (21.4%)
	T4	5 (5.1%)
N stage (%)	N0	45 (45.9%)
	N1	18 (18.4%)
	N2	14 (14.3%)
	N3	6 (6.1%)
M stage (%)	M0	97 (99%)
	M1	1 (1%)
Clinical stage (%)	Stage I	33 (32.7%)
	Stage II	20 (20.4%)
	Stage III	29 (29.6%)
	Stage IV	1 (1%)
Gender (%)	Female	43 (43.9%)
	Male	55 (56.1%)
Age (%)	≤65	70 (71.4%)
	>65	28 (28.6%)
PD-L1 positive	<5%	32 (32.7%)
rate (%)	≥5%	52 (53.1%)
CD8 positive	<5%	27 (27.6%)
rate (%)	≥5%	47 (48%)
Staining score of	≤1	46 (46.9%)
CKS2	>1	52 (53.1%)

## Abbreviations

CKS2: Cyclin-dependent kinase regulatory subunits
LUAD: lung adenocarcinoma
NSCLC: non-small cell lung cancer
LUSC: lung squamous cell carcinoma
OS: overall survival
TCGA: The Cancer Genome Atlas
GEO: Gene Expression Omnibus
GO: gene ontology
KEGG: Kyoto Encyclopedia of Genes and Genomes
GSEA: gene set enrichment analysis
MSigDB: Molecular Signatures Database
NES: normalized enrichment score
FDR: false discovery rate
ssGSEA: single-sample Gene Set Enrichment Analysis
DEGs: differentially expressed genes
Mut: mutation type
Wt: wild type
HR: hazard ratio
CI: confidence interval
FPR: false positive rate
DCs: dendritic cells
ICIs: immune checkpoint inhibitors
